# Association between Serum Bilirubin and Acute Intraoperative Hyperglycemia Induced by Prolonged Intermittent Hepatic Inflow Occlusion in Living Liver Donors

**DOI:** 10.1371/journal.pone.0156957

**Published:** 2016-07-01

**Authors:** Sangbin Han, Sang-Man Jin, Justin Sangwook Ko, Young Ri Kim, Mi Sook Gwak, Hee Jeong Son, Jae-Won Joh, Gaab Soo Kim

**Affiliations:** 1 Department of Anesthesiology and Pain Medicine, Samsung Medical Center, Sungkyunkwan University School of Medicine, Seoul, Korea; 2 Department of Anesthesiology and Pain Medicine, Kangwon National University School of Medicine, Chuncheon, Korea; 3 Division of Endocrinology and Metabolism, Department of Medicine, Samsung Medical Center, Sungkyunkwan University School of Medicine, Seoul, Korea; 4 Department of Surgery, Samsung Medical Center, Sungkyunkwan University School of Medicine, Seoul, Korea; University of Toledo, UNITED STATES

## Abstract

**Background:**

Intermittent hepatic inflow occlusion (IHIO) is associated with acute hyperglycemia during living donor hepatectomy when the ischemia is prolonged. Bilirubin is a potent antioxidant to play an important role for maintaining insulin sensitivity and preventing hyperglycemia. Thus, we aimed to test whether serum bilirubin level is associated with prolonged IHIO-induced intraoperative hyperglycemia.

**Methods:**

Seventy-five living liver donors who underwent a prolonged IHIO with a >30 minute cumulative ischemia were included. The association between preoperative serum bilirubin concentrations and the risk of intraoperative hyperglycemia (blood glucose concentration >180 mg/dl) was analyzed using binary logistic regression with adjusting for potential confounders including age and steatosis.

**Results:**

The number of donors who underwent 3, 4, 5, and 6 rounds of IHIO was 41, 22, 7, and 5, respectively. Twenty-nine (35%) donors developed intraoperative hyperglycemia. Total bilirubin concentration was inversely associated with hyperglycemia risk (odds ratio [OR] 0.033, 95% confidence interval [CI] 0.004–0.313, *P* = 0.003). There was an interaction between age and total bilirubin concentration: the effect of lower serum total bilirubin (≤0.7 mg/dl) on the development of hyperglycemia was greater in older donors (>40 years) than in younger donors (*P* = 0.0.028 versus *P* = 0.212). Both conjugated bilirubin (OR 0.001 95% CI 0.001–0.684) and unconjugated bilirubin (OR 0.011 95% CI 0.001–0.246) showed an independent association with hyperglycemia risk.

**Conclusions:**

Lower preoperative serum bilirubin was associated with greater risk of prolonged IHIO-induced hyperglycemia during living donor hepatectomy particularly in older donors. Thus, more meticulous glycemic management is recommended when prolonged IHIO is necessary for surgical purposes in old living donors with lower serum bilirubin levels.

## Introduction

The severe shortage of available liver transplant grafts has led to the acceptance of living donors. However, living donor hepatectomy has been associated with considerable complications. Our data showed major complication rates of around 7% and overall complication rates of around 35%, including infectious complications and wound problems [1, 2]. Although most of the complications are reversible and short-term, concerns for the safety of healthy donors should be more emphasized to minimize harm to them from the ethical standpoint. More importantly, the mortality of living liver donors has not been zero. In a report published in 2006, there were 19 known deaths (0.15% mortality) and 5 donors of them died due to infectious complications [3].

Intermittent hepatic inflow occlusion (IHIO) technique could be used in living donor hepatectomy to reduce parenchymal bleeding and exposure to allogeneic blood as well as to precondition the graft prior to a period of cold ischemia [4, 5]. However some donors develop acute intraoperative hyperglycemia when the cumulative ischemia time is >30 minutes [6], which is a so-called reperfusion hyperglycemia [7, 8]. Deleterious effects of acute hyperglycemia include the increase in the risk of infectious complication [9, 10], the increase in the extent of hepatic ischemia reperfusion injury [8, 11, 12], and the negation of the benefit of preconditioning [5, 11, 13]. Thus, it is important to estimate the development of hyperglycemia and manage glycemic disturbances in timely manner.

Oxidative stress is an important component of insulin resistance, glucose intolerance, and hyperglycemia. As a potent antioxidant [14], bilirubin plays a key role to maintain insulin sensitivity and insulin secretion, and thus, prevent abrupt hyperglycemic changes [15–17]. This protective effect is known to be dose dependent even in the physiologic ranges shown in the general population [18, 19]. Thus, we tested the value of serum bilirubin as a predictor for prolonged IHIO-induced acute intraoperative hyperglycemia in healthy living donors.

## Methods

### Subjects and data collection

One hundred fifty-four adult living donors who underwent open right hepatectomies between December 2010 and August 2012, when blood glucose concentration was monitored in great detail during IHIO [6, 8], were the initially screened cohort. Among these, 75 donors who underwent a prolonged IHIO (≥3 rounds of IHIOs which is correspondent to >30 minutes cumulative ischemia) during liver resection were included in the study.

Routine pretransplant evaluation included biochemical tests, abdominal ultrasound, computed tomography angiography, and magnetic resonance cholangiopancreatography. Acceptance criteria for liver donation were age ≤65 years, body mass index <35 kg/m^2^, macrosteatosis ≤30% irrespective of microsteatosis [1, 2], and residual liver volume ≥30%. Serum bilirubin concentration did not limit the candidacy for donation *per se* if there were no evidence of hepatobiliary or hematologic diseases since isolated hyperbilirubinemia was clinically diagnosed as benign hereditary hyperbilirubinemia, like Gilbert syndrome, and not thought to increase postoperative complications, including biliary complication [20]. Conjugated and unconjugated bilirubin concentrations were estimated from total and direct bilirubin, being measured using Jendrassik-Grof diazo procedure with caffeine/benzoate solution [20].

The Institutional Review Board of Samsung Medical Center approved this retrospective cohort study (SMC 2015-03-088) and waived the requirement for written informed consent. All data were obtained from ordinary electronic medical record or a prospectively collected liver transplantation database and were anonymized and de-identified prior to analysis. None of the transplant donors were from a vulnerable population and all donors provided written informed consent for the donation of their livers that was freely given.

### Monitoring and anesthesia

Intraoperative anesthetic management was performed according to the standardized protocol for living donor hepatectomy. After standard anesthetic monitoring, morphine sulfate (400 μg) was intrathecally administered to alleviate postoperative pain [21]. General anesthesia was induced with sodium thiopental (5 mg/kg) and maintained with isoflurane. An intra-arterial catheter was placed in the right radial artery for direct blood pressure monitoring and blood sampling. Remifentanil was infused intravenously based on the hemodynamic responses. Vecuronium was used as a muscle relaxant to facilitate muscle relaxation. Ventilation was controlled to obtain a tidal volume of 8–10 ml/kg and maintain normocapnea, and the fraction of inspired oxygen was maintained at 0.4–0.5. Circulation was managed to achieve a target mean arterial pressure of ≥70 mmHg. Hartmann’s solution was primarily administered to maintain normovolemia.

### Operative management

After an acceptable graft quality was histologically confirmed via a wedge liver biopsy, a transection plane was defined by means of a temporary inflow occlusion of the right hepatic artery and portal vein. A cavitron ultrasonic aspirator and bipolar electrocautery were used for liver resection, thereby avoiding crushing by clamps. IHIO was applied liberally in an “on-demand” manner on the basis of the amount of bleeding during parenchymal dissection [5]. During each round of IHIO, the hepatic artery and portal vein were clamped using a vascular tourniquet for 15 minutes and unclamped for 5 minutes. The cut parenchymal surface was assessed during each unclamping period, and the clamping-unclamping maneuver was repeated in a step-by-step fashion if the bleeding was thought to originate mainly from the vascular inflows.

### Glycemic Management Protocol

Donors were instructed to refrain from drinking alcohol or smoking once they were scheduled for surgery (usually for a period of 2 to 4 weeks) and hospitalized 2 days before surgery for a final evaluation including standard liver function tests. They were fasted overnight, and 5% dextrose in normal saline (NaK3 5%, CJ, Seoul, Korea) was infused at a rate of 80 ml/hr during the fasting period. No preoperative oral carbohydrate supplements were provided. Arterial blood glucose concentrations were measured immediately after anesthesia induction and every 2 hours thereafter using a blood gas/chemistry analysis (RAPIDLAB1265^®^, Siemens Healthcare Diagnostics Inc., Berlin, Germany) and the moment of clamping and unclamping for every IHIO using a hand-held point-of-care glucometer analysis (Precision PCx, Abbott Laboratories, Abbott Park, IL). Regular insulin was indicated when serially measured glucose concentrations were >200 mg/dl.

### Variables and Statistical Analysis

The primary outcome was prolonged IHIO-induced acute intraoperative hyperglycemia (blood glucose concentration >180 mg/dl) [8, 22]. In univariable analysis, the Mann Whitney test (continuous variables), and Chi-square test or Fisher's exact test (categorical variables) were used. Analyzed independent variables included age [23], psoas muscle area [24], alcohol consumption [25], smoking history, liver enzyme levels (alanine transaminase and γ-glutamyl transpeptidase) [26], and cumulative ischemia time of IHIO [6]. Psoas muscle area was measured on the right psoas muscle of a single cross-sectional computed tomography scan image at the 3rd lumbar level [27]. Variables with *P* value <0.10 during univariable analysis were entered into binary logistic regression analysis [28]. The Hosmer-Lemeshow test was used to test for goodness of fit for the multivariable model. The risk of multicollinearity was evaluated by means of variance inflation factor. For a subgroup analysis, donors were stratified according to the age of 40 years and serum total bilirubin concentration of 0.7 mg/dl, which was determined using optimum stratification to find the most significant *P* value [29]. As an exploratory analysis, the incidence of surgical site infection was compared according to the presence or absence of intraoperative hyperglycemia. Surgical site infection included superficial/deep incisional infections and organ/space infections [30]. Continuous variables are expressed as medians (25th percentile, 75th percentile). Categorical variables are expressed as frequency (%). The results of the logistic regression analysis are described as the odds ratio (OR) with 95% confidence interval (CI). All reported *P* values were two-sided, and *P* <0.05 was considered statistically significant. SPSS 19.0 (SPSS Inc, Chicago, IL) was used for statistical analyses.

## Results

**Fig 1:**
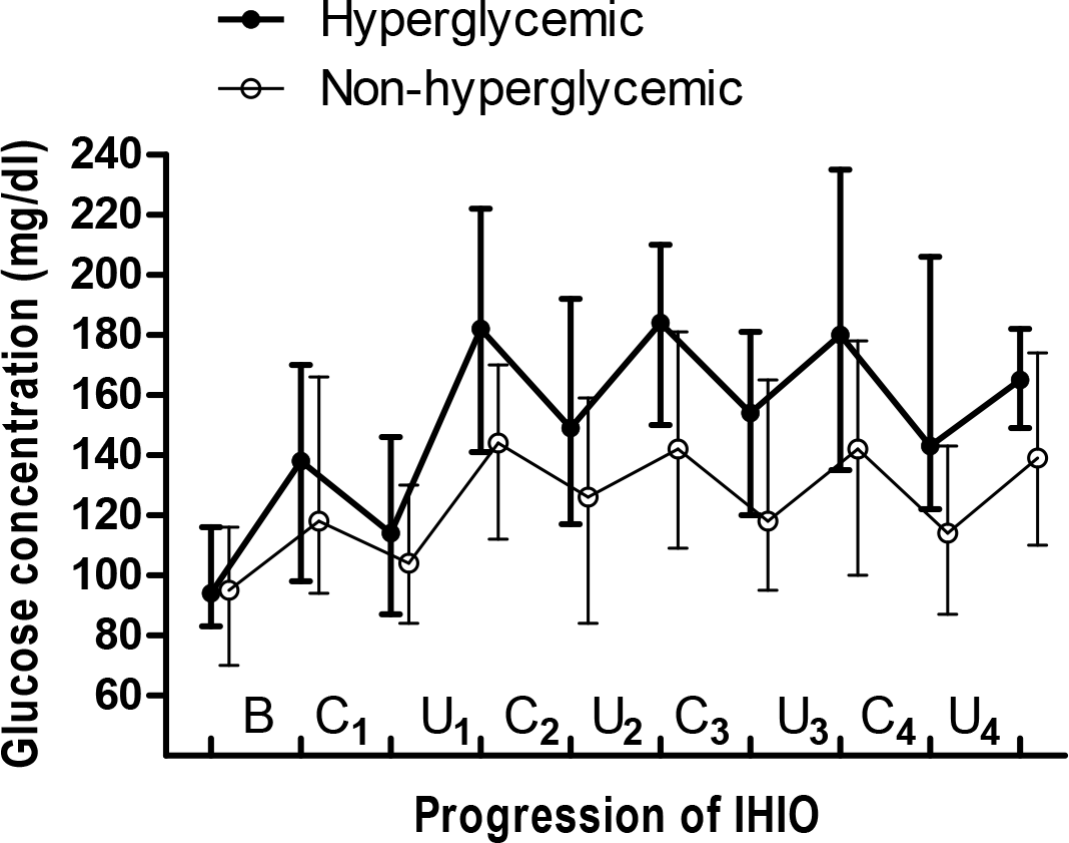
Intraoperative blood glucose concentrations before the start of IHIO (B), during each clamping period (C), and during each unclamping period (U) in donors who developed intraoperative hyperglycemia and in donors without intraoperative hyperglycemia. Circlet indicates the median and whiskers indicate the range.

No donors had a previous diabetes history. The number of donors who underwent 3, 4, 5, and 6 rounds of IHIO was 41, 22, 7, and 5, respectively. There were no donors who received any blood products transfusion or glucose fluids during surgery. The median number of glucose measurement during surgery was 11 (9–13). Twenty-nine (38.7%) donors developed intraoperative hyperglycemia. Dynamic up-and-down glycemic changes in donors with intraoperative hyperglycemia and without intraoperative hyperglycemia are shown in Fig 1.

**Fig 2:**
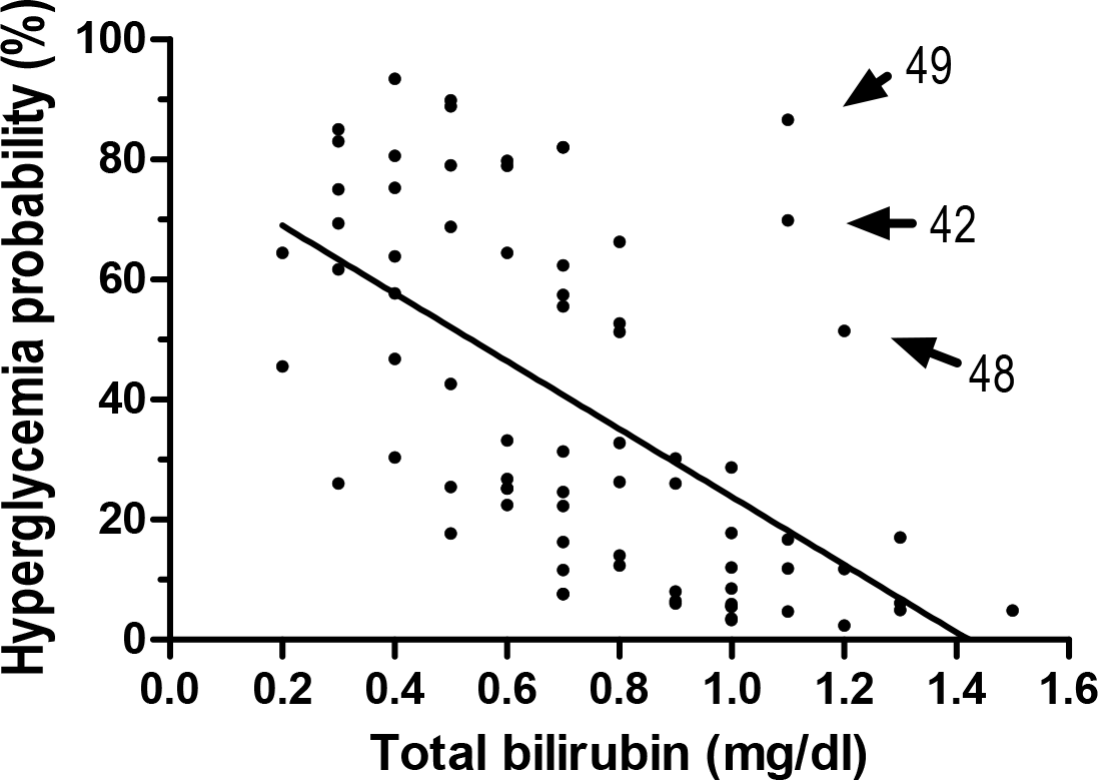
Adjusted probability of intraoperative hyperglycemia in relation to serum total bilirubin level. The number next to the arrow indicates the age (years) of each donor.

In univariable analysis, age and preoperative serum total/unconjugated/conjugated bilirubin concentrations were significantly associated with intraoperative hyperglycemia (*P* <0.05) (Table 1). As correlation coefficients between total, unconjugated, and conjugated bilirubin level were >0.7 (*P* <0.0001), each bilirubin was separately entered into multivariable analysis (Table 2). Goodness of fit for each multivariable model was satisfactory and variance inflation factors for all variables were <2. Serum total bilirubin concentration was inversely associated with the risk of hyperglycemia (OR 0.033, 95% CI 0.004–0.313, *P* = 0.003). Unconjugated bilirubin (OR 0.011, 95% CI 0.001–0.246) and conjugated bilirubin (OR 0.001, 95% CI 0.001–0.684) concentrations were inversely associated with the risk, too. Fig 2 shows the inverse correlation between total bilirubin concentration and the adjusted probability of hyperglycemia (*P* <0.001). As shown in Fig 2, the probability of hyperglycemia of 3 donors clearly deviated from the linear regression line. The age of the three donors was >40 years (42, 48, and 49 years). This finding was consistent with the result of multivariable analysis that older age was independently associated with greater hyperglycemia risk (OR 1.079, 95% CI 0.023–1.139, *P* = 0.005).

**Table 1:** Univariable analysis for detecting predictors for intraoperative hyperglycemia (>180 mg/dl).

	Non-hyperglycemic (n = 46)	Hyperglycemic(n = 29)	*P*
Age (year)	29 (25–35)	42 (30–50)	.001
Female sex	36 (78.3)	18 (62.1)	.128
Body mass index (kg/m^**2**^**)**	23.3 (21.9–25.2)	23.5 (20.9–26.8)	.479
Waist circumference (cm)	82.0 (76.5–86.8)	85.0 (75.0–91.5)	.252
Psoas muscle area (mm^2^)	950 (669–1170)	910 (569–1100)	.508
Non-alcoholics	24 (52.2)	13 (44.8)	.958
Never smoker	28 (60.9)	18 (62.1)	.917
Preoperative biochemical tests			
Fasting glucose (mg/dl)	95 (89–98)	94 (92–99)	.281
Total cholesterol (mg/dl)	176 (162–200)	190 (166–212)	.232
Total bilirubin (mg/dl)	0.8 (0.6–1.0)	0.6 (0.4–0.8)	.003
Unconjugated bilirubin (mg/dl)	0.6 (0.5–0.8)	0.4 (0.3–0.6)	.003
Conjugated bilirubin (mg/dl)	0.2 (0.1–0.3)	0.1 (0.1–0.2)	.008
Alanine transaminase (IU/l)	17 (11–20)	17 (12–25)	.073
γ-glutamyl transpeptidase (IU/l)	16 (11–23)	18 (11–32)	.382
Total steatosis degree > 15%	13 (28.3)	14 (48.3)	.079
Remnant liver volume (%)	35 (33–39)	37 (34–40)	.584
Cumulative ischemia time (minutes)	49 (47–63)	55 (46–63)	.755
Crystalloid (ml/hr)	365 (330–413)	337 (283–412)	.223
Blood loss (ml)	134 (54–228)	153 (47–279)	.600

Data are described as mean ± standard deviation or frequency (%).

**Table 2:** Multivariable analysis for detecting predictors for intraoperative hyperglycemia (>180 mg/dl).

	Odds ratio (95% confidence interval)	*P*
Total bilirubin (mg/dl)	0.033 (0.004–0.313)	.003
Age (years)	1.079 (1.023–1.139)	.005
Alanine transaminase (IU/l)	1.072 (0.986–1.167)	.104
Total steatosis degree >15%	2.708 (0.809–9.060)	.106
Unconjugated bilirubin (mg/dl)	0.011 (0.001–0.246)	.004
Age (years)	1.086 (1.025–1.151)	.005
Alanine transaminase (IU/l)	1.097 (0.990–1.217)	.078
Total steatosis degree >15%	2.572 (0.691–9.576)	.159
Conjugated bilirubin (mg/dl)	0.001 (0.001–0.684)	.041
Age (years)	1.086 (1.026–1.149)	.004
Alanine transaminase (IU/l)	1.048 (0.959–1.146)	.298
Total steatosis degree >15%	2.472 (0.708–0.636)	.156

Variables of *P* <0.10 during univariable analysis were included in the model. Total bilirubin, unconjugated bilirubin, and conjugated bilirubin levels were separately entered into each multivariable model due to strong correlation among them.

**Fig 3:**
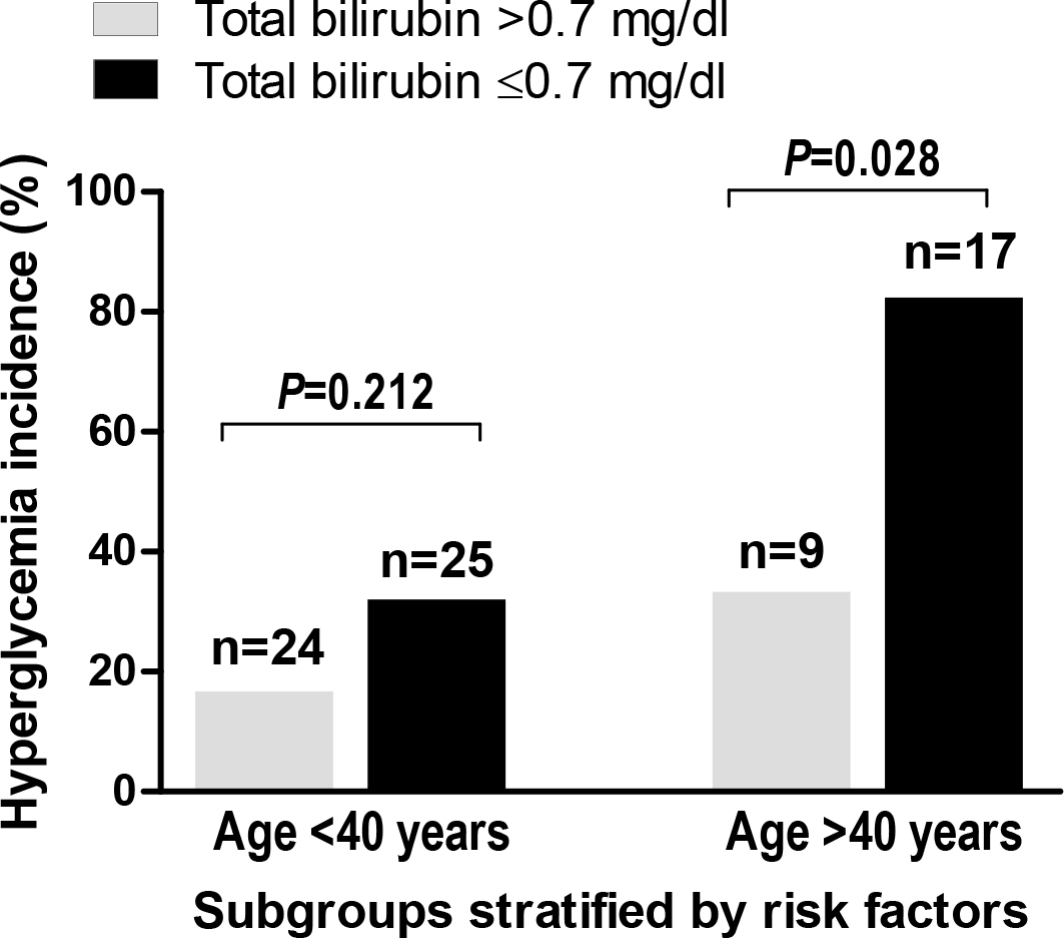
Interaction between serum bilirubin and age. The impact of lower serum bilirubin on the development of intraoperative hyperglycemia is stronger in old donors (>40 years) than in younger donors.

In the subgroup of donors with age of <40 years, the incidence of hyperglycemia was not significantly different between donors with total bilirubin of ≤0.7 mg/dl and donors with total bilirubin of >0.7 mg/dl (32.0% versus 16.7%, *P* = 0.212). In contrast, in the subgroup of donors with age of >40 years, hyperglycemia incidence was significantly greater in donors with total bilirubin of ≤0.7 mg/dl than in donors with total bilirubin of >0.7 mg/dl (82.4% versus 33.3%, *P* = 0.028) (Fig 3).

Postoperative surgical site infection was tended to be higher in donors who developed intraoperative hyperglycemia (3 of 26 hyperglycemic donors [10.3%] versus 2 of 44 non-hyperglycemic donors [4.3%]).

## Discussion

In this study, we analyzed 75 healthy living donors without diabetes who underwent ≥3 rounds of IHIO during right hepatectomy. Blood glucose concentration was measured at every start and end of a IHIO round. Higher Serum bilirubin was independently associated with the decrease in the risk of intraoperative acute hyperglycemia. As the first to open up the possibility of intrinsic metabolism of donor liver affecting their clinical courses, our data suggest that serum bilirubin concentrations could be used as an index for planning glycemic managements such as deciding time points for the measurement of blood glucose level and the initiation of insulin therapy. We also found an interaction between age and bilirubin level. The association between lower serum bilirubin and higher hyperglycemia risk was stronger in donors >40 years old compared with younger donors. Accordingly, the incidence of hyperglycemia in >40 years donors with lower serum bilirubin (≤0.7 mg/dl) reached 82.4% after prolonged IHIOs. Also, there was a trend toward the increase in surgical site infection risk in donors who developed intraoperative hyperglycemia.

Blood glucose level during IHIO rises following each reperfusion (Fig 1). Hepatic inflow occlusion decreases the energy level inside hepatocytes and blocks glucose delivery into hepatocytes, and thus, leads to massive hepatic glycogenolysis. Subsequent reperfusion results in damages to the hepatocytes, which is a so-called ischemia reperfusion injury, and simultaneously, efflux of glucose from injured hepatocytes into the systemic circulation occurs [7]. In our previous study of living donors, it was found that blood glucose concentrations continued increasing in some donors, whereas the concentrations were maintained in similar levels despite repeated ischemia and reperfusion [6, 31]. This individual variation might be attributable to the difference in different intrinsic glucose metabolism. As a potent anti-oxidant, bilirubin is known to play an important role to maintain glycemic homeostasis (insulin sensitivity and insulin secretion) [14–17]. Thus, it could be deduced that different serum bilirubin levels are associated with different glucose metabolism and our data clearly demonstrated the inverse relationship between serum bilirubin level and the risk of IHIO-induced hyperglycemia. The main action of this potent antioxidant bile pigment to prevent IHIO-induced hyperglycemia might be primarily attributable to the difference in intrinsic glucose metabolism rather than the difference in the extent of hepatocytes injury or the amount of hepatic glucose efflux because no correlations were found between preoperative total bilirubin concentration and hepatocytes injury indices (maximum postoperative aspartate transaminase [*P* = 0.582] and alanine transaminase [*P* = 0.582], respectively).

Living donor safety is an issue of cardinal significance and it should be avoided to expose donors to unnecessary risk. Our study offers a guideline for avoiding hyperglycemia by means of timely glycemic measurement and insulin therapy instead of delayed recognition. Hyperglycemia does not require prolonged exposure or underlying diabetes to play its deleterious effects. Experimental research has shown that acute hyperglycemia for a transient period increased ischemia reperfusion injury of the liver and impaired microcirculation [11, 32]. Clinical evidence of patients undergoing hepatectomy has shown the association between acute intraoperative hyperglycemia and hepatocytes injury [8, 12]. Also, acute hyperglycemia is known to abolish protective effect of ischemic preconditioning [11, 13]. In our recent study, recipient survival benefit associated with IHIO during living donor hepatectomy disappeared when the cumulative ischemia time was >30 minutes, and prolonged IHIO-induced hyperglycemia was suspected to be one of underlying mechanisms [5].

Healthy living donors who underwent full preoperative screening process were supposed to have homogenous general conditions. Accordingly, biasing effects of preexisting diseases which impair glycemic homeostasis, such as diabetes, cirrhosis, pancreatitis, and acute illness, could be minimized. Predetermined and standardized nature of surgical procedures, the lack of inadvertent surgical incursions (massive bleeding, rapid fluid infusion, and transfusion), and the lack of major anesthetic events (hypoxemia, hypothermia, and catecholamine infusion) further added robustness of our data. Moreover, the stable intraoperative condition is known to increase the accuracy of the point-of-care glucometer [33].

The respective importance of unconjugated and conjugated bilirubin remains unclear. While most studies focused on unconjugated bilirubin, some have suggested the equality in antioxidant effects of the two bile pigments [34, 35]. We could not clearly answer this issue because preoperative unconjugated and conjugated bilirubin concentrations were strongly correlated (coefficient 0.70, *P* <0.001) each other. At least, total bilirubin level could be used as a surrogate marker reflecting the effect of unconjugated or conjugated bilirubin, or sum of them because total bilirubin was strongly correlated with unconjugated bilirubin (coefficient 0.98) and conjugated bilirubin(coefficient 0.82) (*P* <0.001, each).

The retrospective nature limits the interpretation of our findings. Although the relationship between serum bilirubin and insulin sensitivity is already well-known, insulin measurement and the calculation of homeostatic model assessment (HOMA), which is a surrogate marker for insulin sensitivity, might have refined our findings. A limited sample size is an additional disadvantage. Particularly, the sample size was insufficient to perform statistical analysis regarding postoperative complications relevant to the current study (e.g. surgical site infection). Indeed, we could not even prospectively gain more donors undergoing prolonged IHIO because short IHIOs has been a general practice in our transplant team, instead of the previous liberal strategy, since our data demonstrated the safety and recipient survival benefit of 1–2 rounds of IHIOs and the negative effects of ≥3 rounds of IHIOs [5, 31, 36].

Although IHIO technique has been accepted as an effective method to reduce liver parenchymal blood loss with a potential benefit of graft preconditioning, there have been no clinically relevant evidence for the management of IHIO-induced glycemic disturbances. Our data show that lower serum bilirubin is an independent predictor for acute intraoperative hyperglycemia after prolonged IHIO. The risk of lower serum bilirubin is more significant when donor age is >40 years. Thus, more meticulous glucose measurement is recommended for optimal glycemic management when prolonged IHIO is necessary for surgical purposes particularly in old living donors with lower serum bilirubin levels.

## Abbreviations

HOMA: homeostatic model assessment
IHIO: intermittent hepatic inflow occlusion
OR: odds ratio
CI: confidential interval
